# A Rare Case of Non-traumatic Tension Pneumocephalus Following a Lumbar Puncture: Unusual Complications of a Common Procedure

**DOI:** 10.7759/cureus.82888

**Published:** 2025-04-24

**Authors:** Wilson Ong, Ren Wei Liu, Xi Zhen Low, Pin Lin Kei, David Daoyong Lai

**Affiliations:** 1 Radiology, National University Hospital, Singapore, SGP; 2 Radiology/Neuroradiology, National University Hospital, Singapore, SGP; 3 Radiology/Neuroradiology, Ng Teng Fong General Hospital, Singapore, SGP

**Keywords:** cerebrospinal fluid leak, lateral sphenoid encephalocele, lumbar puncture, skull base defect, tension pneumocephalus

## Abstract

Tension pneumocephalus is a rare but severe complication characterized by intracranial air accumulation, leading to increased intracranial pressure (ICP). While it is most commonly associated with trauma, surgical interventions, tumors, or infections, spontaneous cases related to skull base defects and cerebrospinal fluid (CSF) leaks are uncommon. We present what we believe to be one of the first reported cases of tension pneumocephalus following a diagnostic lumbar puncture (LP) in an otherwise healthy individual with no predisposing history, prior history of skull base trauma or surgery. The patient was a 66-year-old female who presented with altered mental status. Initial non-contrast CT brain scans showed no evidence of pneumocephalus, though incidental left sphenoid sinus opacification was noted. Following the LP, the patient’s neurological symptoms worsened, and subsequent imaging revealed tension pneumocephalus. Further evaluation confirmed a focal dehiscence in the posterolateral wall of the left sphenoid sinus, with herniation of brain tissue consistent with a lateral sphenoid encephalocele. We hypothesize that the LP induced a negative ICP gradient, precipitating a CSF leak and ingress of air through the skull base defect. As intracranial air pressure increased, the encephalocele likely functioned as a one-way "ball-valve" mechanism, exacerbating the tension pneumocephalus. This case highlights the potential for routine diagnostic procedures to result in serious complications in patients with undiagnosed skull base defects. Detecting subtle encephaloceles and areas of skull base dehiscence can be difficult on non-contrast imaging, emphasizing the need for high clinical suspicion, especially when evaluating for CSF leaks. Multidisciplinary management and early recognition of such anatomical vulnerabilities are critical in preventing life-threatening complications associated with tension pneumocephalus.

## Introduction

Pneumocephalus, or intracranial air accumulation, typically arises after traumatic head injuries involving craniofacial or air sinus fractures, surgical interventions, or, in rare instances, cerebrospinal fluid (CSF) drainage [1,2]. Generally, intracranial air accumulation does not lead to significant mass effect or neurological symptoms and often resolves with conservative management [3]. However, in rare and severe cases, tension pneumocephalus may develop, characterized by a one-way valve mechanism that allows air to enter but prevents its exit, leading to increased intracranial pressure (ICP) [4]. This condition can cause mass effect and progressive neurological deterioration. The concept of tension pneumocephalus was first described in 1914 and presents with symptoms similar to elevated ICP, such as severe headache, vomiting, and even loss of consciousness [5]. A hallmark radiographic sign is the "Mount Fuji" appearance on computed tomography (CT), first noted by Ishiwata et al. [6], typically involving subdural air collection compressing the frontal lobes. In this report, we describe a rare case of tension pneumocephalus following a diagnostic lumbar puncture in a patient who has no known history of trauma or previous surgery, highlighting its unique pathophysiology and clinical challenges.

## Case presentation

A 66-year-old woman presented to the emergency department with altered mental status following a week of low-grade fever without specific localizing symptoms. She experienced a transient generalized tonic-clonic seizure, managed with IV lorazepam. She appeared confused on examination, with a Glasgow Coma Scale (GCS) score of 13. On initial clinical examination, the patient did not exhibit overt meningeal signs - there was no neck stiffness, and Kernig's and Brudzinski’s signs were negative. Blood tests (Table 1) showed leukocytosis (18.1 x 10^9^/L) with neutrophil predominance, metabolic acidosis (anion gap of 26.0 mmol/L, bicarbonate of 17 mmol/L), and markedly elevated liver enzymes (alanine aminotransferase (ALT) of 1,626 U/L, aspartate aminotransferase (AST) of 2,153 U/L), along with mildly elevated bilirubin and high serum lactate (5.3 mmol/L). Serum ammonia levels were within normal limits. These findings raised suspicion for sepsis-related encephalopathy or hepatic dysfunction; however, the normal serum ammonia level and subsequent negative infectious workup made these less likely. This guided the team toward structural causes for her altered mental state, prompting further imaging and investigation.

**Table 1:** Blood test results, which were performed for the patient, and the respective reference ranges.

Test	Result	Normal Range
Full Blood Count		
White Blood Cells (x 10^9^/L)	18.11	4.30–10.40
Red Blood Cells (x 10^12^/L)	4.47	4.03–5.19
Haemoglobin (g/dL)	11.9	11.5–14.9
Haematocrit (%)	37.1	35.8–45.2
MCV (fL)	83.0	80.6–96.1
MCH (pg)	26.6	26.1–32.1
MCHC (g/dL)	32.1	30.8–34.9
RDW (%)	14.6	11.5–14.5
Platelets (x 10^9^/L)	372	150–410
Neutrophils (%)	92.7	-
Neutrophils Absolute (x 10^9^/L)	16.81	1.90–6.53
Lymphocytes (%)	2.8	-
Lymphocytes Absolute (x 10^9^/L)	0.50	1.21–3.56
Monocytes (%)	4.1	-
Monocytes Absolute (x 10^9^/L)	0.74	0.23–0.82
Eosinophils (%)	0.2	-
Eosinophils Absolute (x 10^9^/L)	0.03	0.05–0.09
Basophils (%)	0.2	-
Basophils Absolute (x 10^9^/L)	0.03	0.02–0.09
MPV (fL)	8.6	8.7–12.1
Renal Panel		
Sodium, Serum (mmol/L)	136	135–145
Potassium, Serum (mmol/L)	4.0	3.5–5.2
Chloride, Serum (mmol/L)	97	95–110
Bicarbonate, Serum (mmol/L)	17	22–32
Urea, Serum (mmol/L)	7.4	3.5–7.2
Anion Gap (mmol/L)	26.0	8.0–16.0
Creatinine, Serum (umol/L)	77	45–90
eGFR (mL/min/1.73 m^2^)	70	>90
Liver Function Test		
Albumin	44	32–46
Total Protein, Serum	89	60–80
Bilirubin, Total	57	1–20
Bilirubin, Direct	45	<9
Alanine Transaminase, ALT (U/L)	1626	6–35
Aspartate Transaminase, AST (U/L)	2153	6–30
Alkaline Phosphatase (U/L)	634	46–122
Lactate, Serum (mmol/L)	5.3	<2.0
Ammonia (umol/L)	44	18–72

The initial impression was sepsis of unknown origin, complicated by severe hepatocellular transaminitis and lactic acidosis. She was admitted to the high dependency unit for further evaluation and management. Subsequent CT of the abdomen and pelvis, along with magnetic resonance cholangiopancreatography (MRCP), indicated a likely hepatobiliary source (HBS) of sepsis, with findings suggestive of acute cholecystitis, cholangitis, and choledocholithiasis. She underwent emergent percutaneous cholecystostomy and endoscopic retrograde cholangiopancreatography (ERCP) with plastic biliary stent insertion. Intravenous antibiotics (ceftriaxone and metronidazole) were initiated to treat her underlying sepsis.

**Figure 1:**
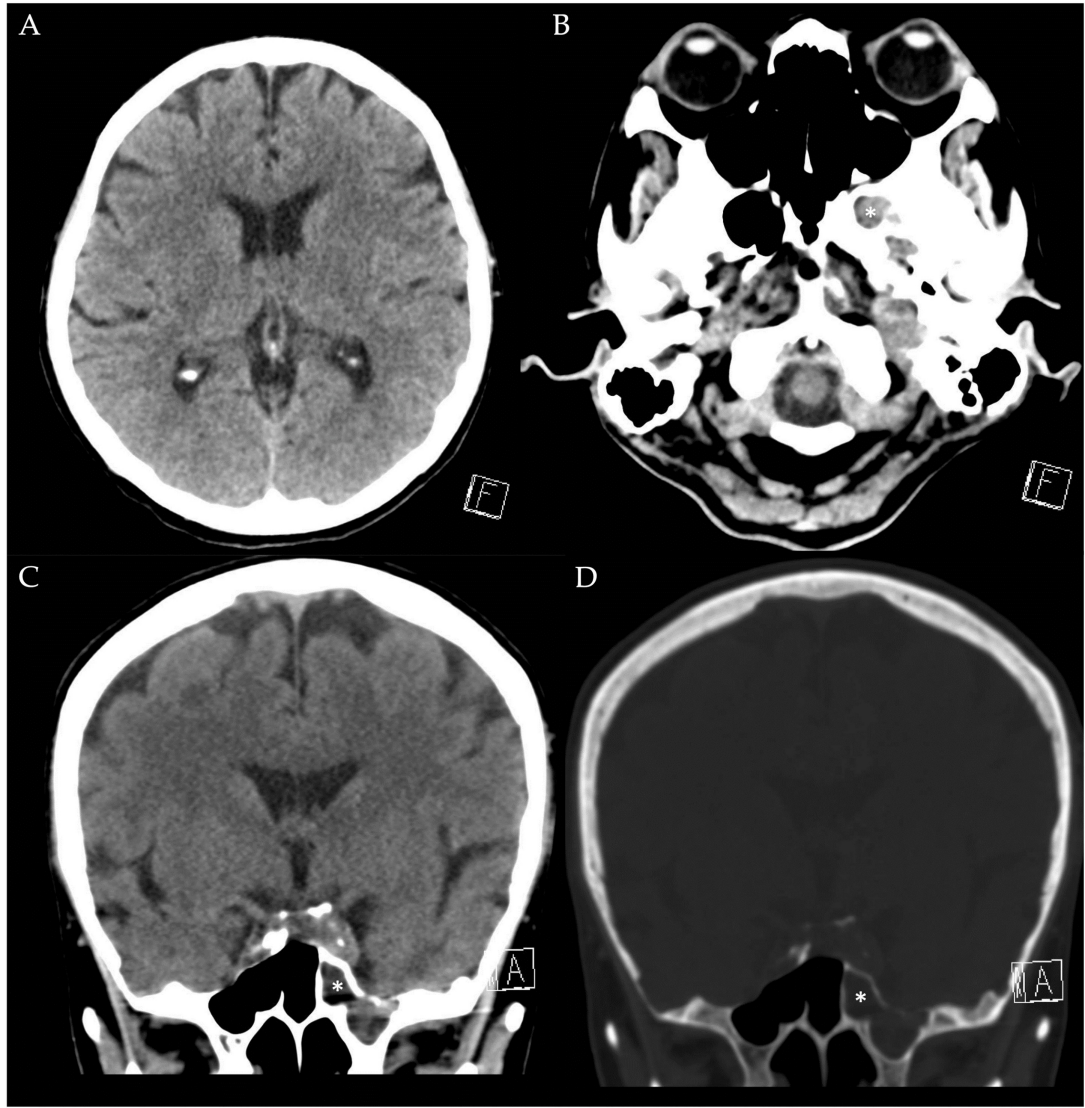
Non-contrast CT brain. 1A (Axial, soft tissue window), showing no significant intracranial abnormality on admission; 1B (axial, soft tissue window); 1C (coronal, soft tissue window); and 1D (coronal, bone window) showing sphenoid sinus opacification (white asterisk).

A non-contrast CT brain scan was performed to evaluate her altered mental status, revealing no significant intracranial findings (Figure 1A), but an incidental left sphenoid sinus opacification (Figures 1B-1D). Based on the clinical suspicion of septic encephalopathy with a need to exclude meningitis in view of persistent altered mental status despite adequate source control and antibiotic therapy for the HBS sepsis, a bedside lumbar puncture was attempted. Unfortunately, the lumbar puncture was unsuccessful despite multiple attempts, and the procedure was aborted. A large amount of clear fluid was noted from her nostrils following the procedure, but it was initially deemed not clinically significant as she had no identifiable risk factors for a CSF leak.

**Figure 2:**
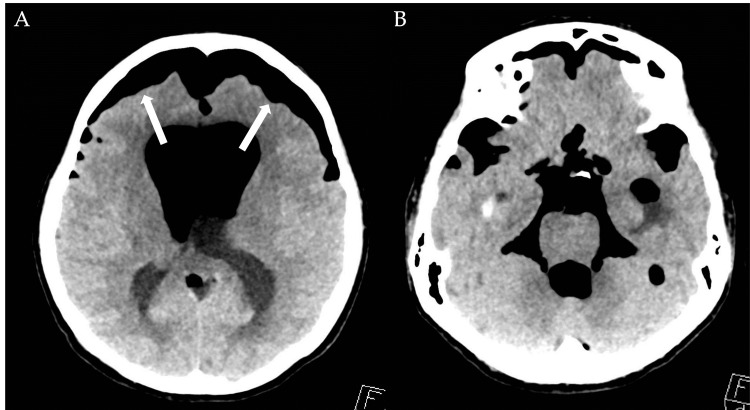
Non-contrast CT brain in the axial cuts, showing extensive pneumocephalus with air in the subarachnoid space and intraventricular space along with “twin-peak” sign (white box arrows), suggestive of tension pneumocephalus.

The patient was initially planned for further evaluation with an MRI of the brain and an electroencephalogram (EEG) for the investigation of his seizure. However, over the next three days, the patient’s mental status gradually worsened. A repeat CT brain scan revealed a large amount of air within the extra-axial brain and intraventricular system, showing the "twin-peak sign," suggestive of tension pneumocephalus (Figures 2A-2B). A small amount of intraventricular hemorrhage was also observed. Examination of the lumbar puncture site showed no significant subcutaneous emphysema to suggest a source of the pneumocephalus.

**Figure 3:**
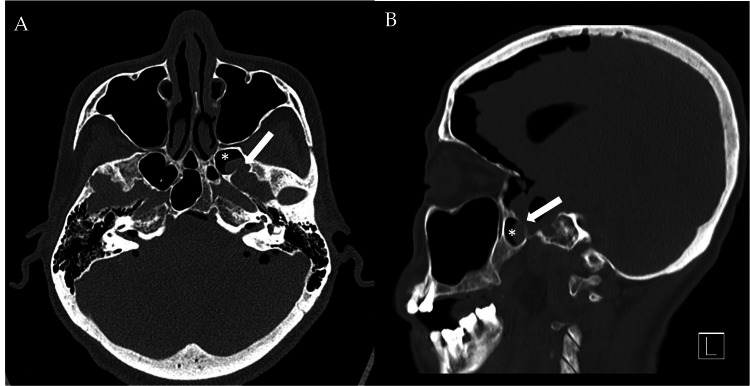
Non-contrast CT skull base in axial (3A) and sagittal (3B) showing focal bony dehiscence (white box arrow) in the posterolateral wall of the left sphenoid sinus (white asterisk) with density protruding within possibly representing encephalocele.

**Figure 4:**
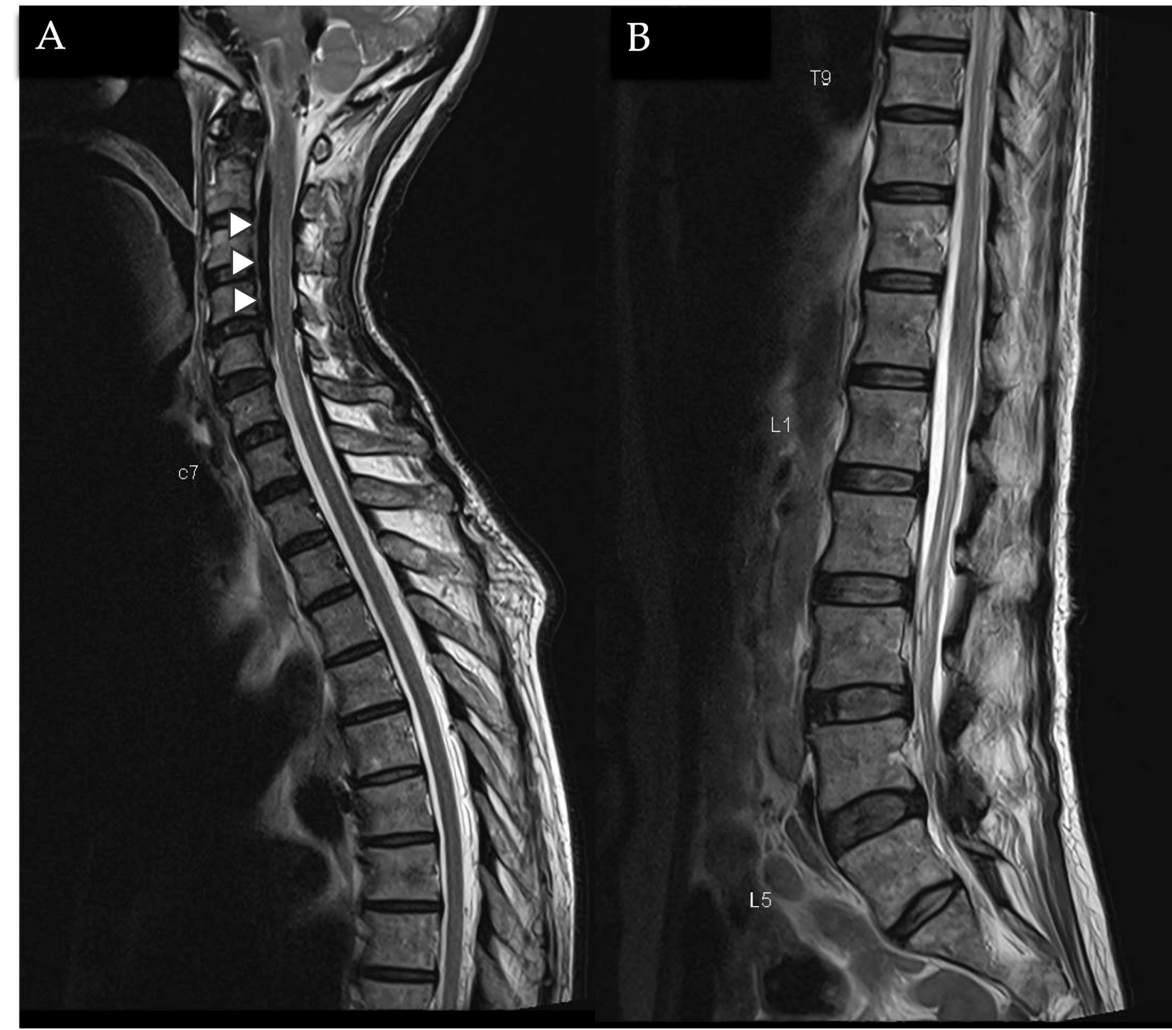
MRI whole spine T2 sequences in sagittal cuts of the cervicothoracic spine (4A) and thoracolumbar spine (4B), showing the presence of air within the epidural space of the cervical canal (white arrowheads), which appears as linear low T2 signal within the epidural space. There is no significant abnormality at the site of the lumbar puncture.

A dedicated CT skull base was performed to investigate the suspected CSF leak and confirmed a focal dehiscence in the posterolateral wall of the left sphenoid sinus, with possible encephalocele (Figures 3A-3B). An MRI of the whole spine was performed to exclude a spinal source of the CSF leak, revealing air within the cervical epidural space but no definite evidence of a CSF leak at the lumbar puncture site (Figures 4A-4B).

**Figure 5:**
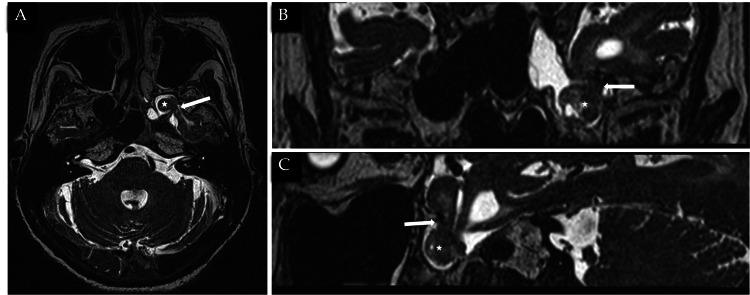
MRI T2 sequences in the axial (5A), coronal (5B), and sagittal (5C), showing brain parenchymal herniating through the defect along the posterolateral sphenoid sinus wall (white arrow) and resultant encephalocele (white star).

**Figure 6:**
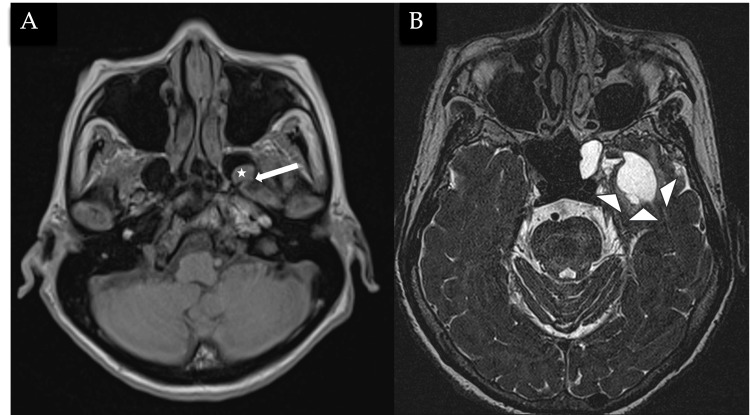
MRI T1 sequences in the axial (6A) showing encephalocele (white star) within the left sphenoid sinus, secondary to a defect in the left posterolateral sphenoid sinus wall (white box arrow). Axial T2 sequences (6B) showing cystic gliosis/encephalomalacia changes in the left temporal lobe (white arrowheads), probably sequelae of chronic herniation.

MRI brain and skull base confirmed the presence of a bony defect along the posterolateral wall of the left sphenoid sinus with part of the temporal lobe herniated (Figures 5A-5C) through the defect and surrounding fluid signal within the left sphenoid sinus. Cystic gliosis/encephalomalacia changes are also seen in the left temporal lobe (Figures 6A-6B). Overall imaging features are in keeping with CSF leak secondary to a left lateral sphenoid encephalocele, with tension pneumocephalus likely precipitated by diagnostic lumbar puncture.

The patient was placed on frequent neurological monitoring, with a plan for urgent decompressive craniotomy should her neurological status deteriorate further. An ENT consultation was obtained, and she subsequently underwent surgical repair of the encephalocele in the left lateral recess. She recovered well post-operatively, with no significant neurological impairment after recovery.

## Discussion

**Figure 7:**
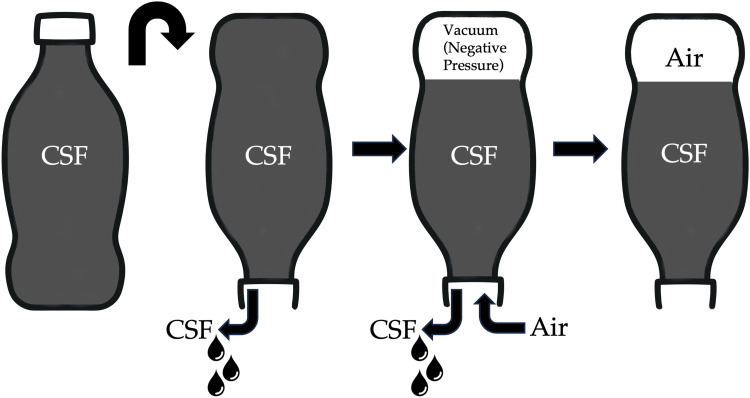
Diagram showing “inverted soda bottle” of pneumocephalus. During a CSF leak from a dural defect, a negative pressure is created in the intracranial system, which results in atmospheric air entering to replace the vacuum, resulting in pneumocephalus. The process will continue until the outside pressure equalizes with the intracranial pressure. Illustrated by: Dr. Wilson Ong

**Figure 8:**
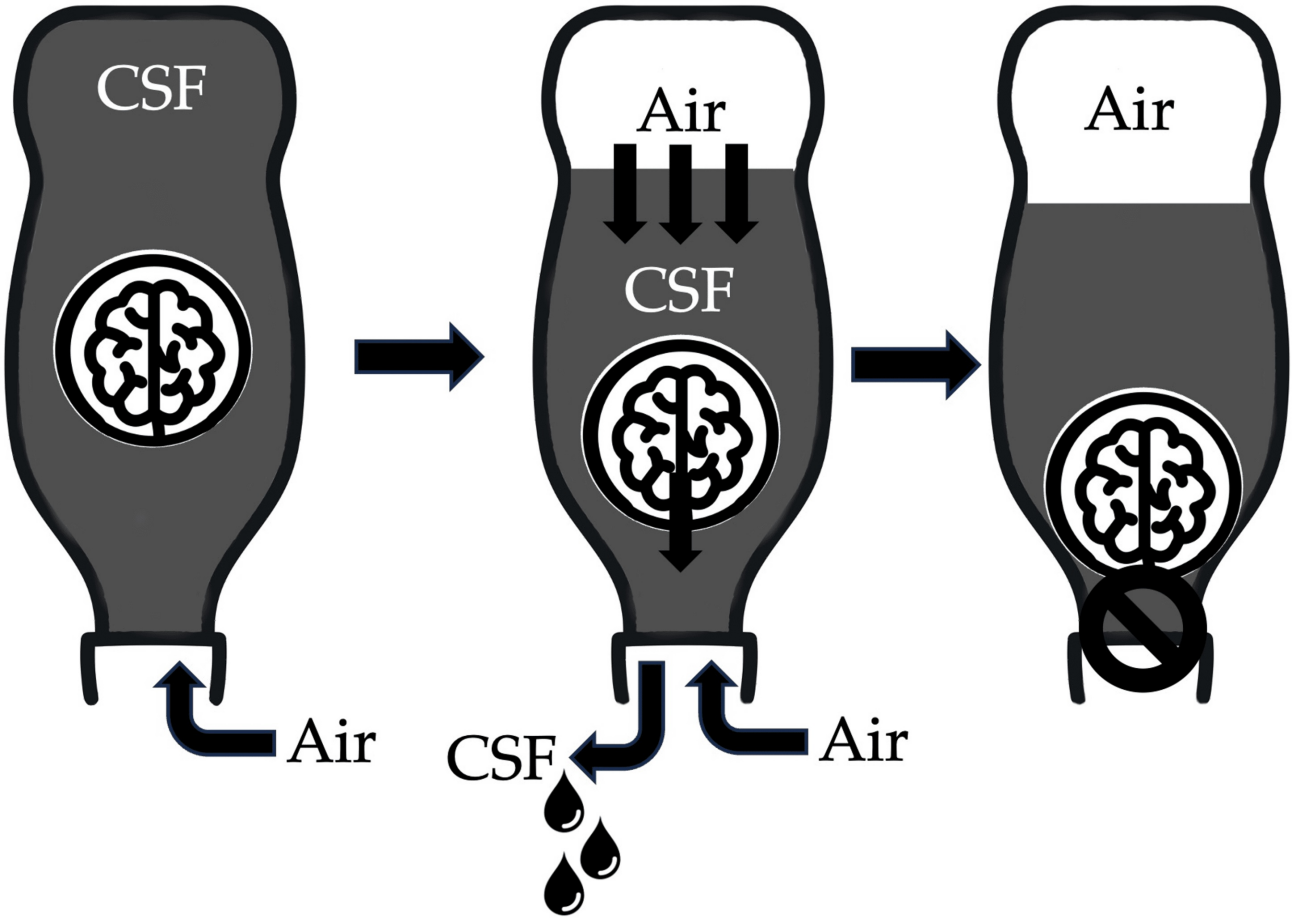
Diagram showing “ball-valve” mechanism of pneumocephalus. Air enters the intracranial system through the dural defect. Increased pressure eventually pushes down on the brain, causing the brain to close the defect so the air cannot escape. Illustrated by: Dr. Wilson Ong

Pneumocephalus is defined as a pathological collection of gas within the cranial cavity, accumulating in the epidural, subdural, subarachnoid, intraventricular, or intraparenchymal compartments [7]. In pneumocephalus, intracranial air can accumulate and lead to either of two mechanisms: the "inverted soda bottle" type, where air entry and exit are slow and self-limiting [3], and the "ball-valve" type [8]. In the “inverted soda bottle” theory (Figure 7), drainage of CSF leads to a negative ICP gradient, which is relieved by the influx of air [9]. The amount of air is independent of the size of the defect, but smaller defects are more easily sealed by blood clots or granulation, allowing for gradual reabsorption and spontaneous resolution of the pneumocephalus [10]. In the “ball-valve” theory (Figure 8), positive pressure events, such as sneezing, coughing, and valsalva maneuvers, force air through a cranial defect. The increase in ICP then causes the defect to be plugged either by meninges or brain parenchyma, which then resists the spontaneous egress of the air. This results in a one-way valve mechanism that causes air to be trapped within the cranial cavity, creating a tension effect that increases ICP. While the common causes of pneumocephalus include trauma, surgical interventions, tumors, and infections [11], spontaneous cases are notably rare and are often linked to CSF leaks due to skull base defects [12].

The most common locations for skull base defects, resulting in spontaneous CSF leak, are the tegmen tympani and the anterior cranial fossa (particularly along the cribiform plate and the lateral process of the sphenoid sinus) [10], where meningeal tissue may adhere directly to the bone, making these sites particularly susceptible to CSF leaks and subsequent air entry [13]. The underlying pathophysiology often involves persistently elevated ICP or intracranial hypertension, which leads to gradual remodeling, thinning, and weakening of the bone at the skull base [14]. Over time, this pressure-driven remodeling creates a defect large enough to allow brain parenchyma to herniate, resulting in an encephalocele [15]. These cases tend to have a predilection for female sex, middle age, and obesity [16] and are frequently associated with “empty-sella sign” on CT [17] as a feature of elevated ICP.

Lateral sphenoid sinus meningoceles, meningoencephloceles, and encophaloceles are relatively rare developing abnormalities that can be seen in patients with chronically elevated intracranial/CSF pressure (such as that seen in idiopathic intracranial hypertension), causing gradual erosion of the sphenoid roof and allowing herniation of the inferior temporal lobe into the lateral sphenoid recess [18]. The lateral sphenoid recess, if pneumatized, is bounded by the foramen rotundum superiorly, the internal carotid posteriorly, the pterygopalatine fossa anteriorly, and the Vidian canal inferiorly and may be surgically challenging to fix [19].

In our case, the patient presented with a lateral sphenoid encephalocele, which was likely incidental and initially asymptomatic. We postulate that the lumbar puncture performed to evaluate her altered mental status may have precipitated a negative ICP state (as in inverted soda bottle type), causing the encephalocele to retract and triggering a CSF leak and rhinorrhea. This created a pathway for air to enter the cranial cavity, leading to pneumocephalus. As ICP increased due to the accumulating air, the encephalocele likely acted as a "ball-valve" mechanism, sealing the defect along the posterolateral sphenoid wall and resulting in tension pneumocephalus. This sequence underscores the complexity and potential severity of even seemingly routine diagnostic procedures in the context of unrecognized skull base defects.

This case is particularly unique because the patient’s initial CT brain scan revealed no evidence of pneumocephalus, yet tension pneumocephalus developed following the lumbar puncture. This suggests that the procedure triggered a CSF leak, likely due to an undiagnosed skull base defect. Detecting skull base dehiscence and encephaloceles on non-contrast CT brain scans can be challenging, as this imaging modality is primarily used to rule out acute intracranial pathology and assess for signs of raised ICP before performing a lumbar puncture. In the absence of clinical suspicion for CSF rhinorrhea or a leak, subtle skull base abnormalities may be overlooked, especially since encephaloceles can mimic sinus opacification, as seen in conditions like sinusitis or nasal polyps [20]. The presence of radiologic indicators such as the "empty sella sign" on CT may also offer additional clues, along with the appropriate clinical demographics can help raise suspicion for skull base defects in the appropriate clinical context. This underscores the critical importance of recognizing anatomical vulnerabilities that may become clinically significant during routine diagnostic procedures. The initial biochemical abnormalities, though striking, were determined to be secondary to the hepatobiliary source of sepsis and did not correlate with the degree of neurological decline. This clinical-imaging discordance further prompted further evaluation and neuroimaging.

One limitation of this case is the lack of biochemical confirmation of CSF rhinorrhea through laboratory testing. Although clear nasal discharge was observed following the attempted lumbar puncture, it was not tested with a CSF panel such as β-2 transferrin or β-trace protein, as CSF leakage was not initially suspected due to the absence of trauma, prior neurosurgical or ENT intervention, or known skull base pathology. In retrospect, such testing would have been useful in confirming the presence of CSF and further supporting the proposed mechanism of intracranial air ingress via a skull base defect. This highlights the diagnostic challenge in patients with occult encephaloceles and emphasizes the need for heightened clinical suspicion when evaluating unexplained rhinorrhea, particularly following procedures that alter ICP.

## Conclusions

In conclusion, this case highlights a rare and complex presentation of tension pneumocephalus following a diagnostic lumbar puncture in the context of an underlying lateral sphenoid encephalocele. The absence of initial pneumocephalus on CT imaging underscores the challenges in detecting subtle skull base defects, especially when there is no clinical suspicion for a CSF leak. The development of tension pneumocephalus following lumbar puncture illustrates how routine diagnostic procedures can precipitate severe complications in patients with undiagnosed anatomical vulnerabilities. This case emphasizes the importance of thorough evaluation and heightened clinical awareness of skull base defects, particularly in a certain group of patient demographics. Prompt recognition and multidisciplinary management remain critical to preventing and mitigating potentially life-threatening sequelae of such rare conditions.
